# Supplementary material for: Pyruvate Dehydrogenase Kinase 1 inhibition mediated oxidative phosphorylation enhancement in cartilage promotes osteoarthritis progression
Source: BMC Musculoskelet Disord. 2023 Jul 20;24:597. doi: 10.1186/s12891-023-06585-6 (PMC10357736; doi:10.1186/s12891-023-06585-6)
Supplement: Supplementary file 1 — Additional file 1. [file 12891_2023_6585_MOESM1_ESM.zip › MF.Gsea.1653627316242/GOMF_THIOLESTER_HYDROLASE_ACTIVITY.html]

Details for gene set GOMF\_THIOLESTER\_HYDROLASE\_ACTIVITY[GSEA]

|  || Dataset | 55457\_collapsed\_to\_symbols.cls.cls #OA\_versus\_normal.cls.cls #OA\_versus\_normal\_repos |
| Phenotype | cls.cls#OA\_versus\_normal\_repos |
| Upregulated in class | OA |
| GeneSet | GOMF\_THIOLESTER\_HYDROLASE\_ACTIVITY |
| Enrichment Score (ES) | 0.42959553 |
| Normalized Enrichment Score (NES) | 1.4873196 |
| Nominal p-value | 0.03712297 |
| FDR q-value | 0.21626125 |
| FWER p-Value | 1.0 |
Table: GSEA Results Summary

  

Fig 1: Enrichment plot: GOMF\_THIOLESTER\_HYDROLASE\_ACTIVITY      
 Profile of the Running ES Score & Positions of GeneSet Members on the Rank Ordered List

  

| SYMBOL | TITLE | RANK IN GENE LIST | RANK METRIC SCORE | RUNNING ES | CORE ENRICHMENT || 1 | ACOT13 | acyl-CoA thioesterase 13 [Source:HGNC Symbol;Acc:HGNC:20999] | 51 | 0.989 | 0.0964 | Yes |
| 2 | ABHD10 | "abhydrolase domain containing 10, depalmitoylase [Source:HGNC Symbol;Acc:HGNC:25656]" | 437 | 0.690 | 0.1365 | Yes |
| 3 | UFSP2 | UFM1 specific peptidase 2 [Source:HGNC Symbol;Acc:HGNC:25640] | 475 | 0.670 | 0.2016 | Yes |
| 4 | ESD | esterase D [Source:HGNC Symbol;Acc:HGNC:3465] | 529 | 0.647 | 0.2632 | Yes |
| 5 | ACOT7 | acyl-CoA thioesterase 7 [Source:HGNC Symbol;Acc:HGNC:24157] | 971 | 0.524 | 0.2821 | Yes |
| 6 | PPT1 | palmitoyl-protein thioesterase 1 [Source:HGNC Symbol;Acc:HGNC:9325] | 1042 | 0.508 | 0.3283 | Yes |
| 7 | ACOT8 | acyl-CoA thioesterase 8 [Source:HGNC Symbol;Acc:HGNC:15919] | 1088 | 0.500 | 0.3755 | Yes |
| 8 | HAGH | hydroxyacylglutathione hydrolase [Source:HGNC Symbol;Acc:HGNC:4805] | 1395 | 0.443 | 0.3967 | Yes |
| 9 | HIBCH | 3-hydroxyisobutyryl-CoA hydrolase [Source:HGNC Symbol;Acc:HGNC:4908] | 1526 | 0.424 | 0.4296 | Yes |
| 10 | OLAH | oleoyl-ACP hydrolase [Source:HGNC Symbol;Acc:HGNC:25625] | 2673 | 0.283 | 0.3693 | No |
| 11 | ACAA2 | acetyl-CoA acyltransferase 2 [Source:HGNC Symbol;Acc:HGNC:83] | 2779 | 0.272 | 0.3887 | No |
| 12 | ABCD2 | ATP binding cassette subfamily D member 2 [Source:HGNC Symbol;Acc:HGNC:66] | 3031 | 0.246 | 0.3942 | No |
| 13 | PTPRT | protein tyrosine phosphatase receptor type T [Source:HGNC Symbol;Acc:HGNC:9682] | 3157 | 0.234 | 0.4083 | No |
| 14 | ACOT2 | acyl-CoA thioesterase 2 [Source:HGNC Symbol;Acc:HGNC:18431] | 4046 | 0.152 | 0.3547 | No |
| 15 | ABHD17A | "abhydrolase domain containing 17A, depalmitoylase [Source:HGNC Symbol;Acc:HGNC:28756]" | 4733 | 0.093 | 0.3109 | No |
| 16 | ACOT9 | acyl-CoA thioesterase 9 [Source:HGNC Symbol;Acc:HGNC:17152] | 5451 | 0.037 | 0.2589 | No |
| 17 | ACSBG2 | acyl-CoA synthetase bubblegum family member 2 [Source:HGNC Symbol;Acc:HGNC:24174] | 5970 | -0.002 | 0.2189 | No |
| 18 | ABHD17B | "abhydrolase domain containing 17B, depalmitoylase [Source:HGNC Symbol;Acc:HGNC:24278]" | 7410 | -0.112 | 0.1185 | No |
| 19 | ACOT11 | acyl-CoA thioesterase 11 [Source:HGNC Symbol;Acc:HGNC:18156] | 7746 | -0.137 | 0.1064 | No |
| 20 | ABCD3 | ATP binding cassette subfamily D member 3 [Source:HGNC Symbol;Acc:HGNC:67] | 8877 | -0.223 | 0.0412 | No |
| 21 | ABCD1 | ATP binding cassette subfamily D member 1 [Source:HGNC Symbol;Acc:HGNC:61] | 8930 | -0.226 | 0.0601 | No |
| 22 | LYPLA1 | lysophospholipase 1 [Source:HGNC Symbol;Acc:HGNC:6737] | 9023 | -0.234 | 0.0767 | No |
| 23 | PPT2 | palmitoyl-protein thioesterase 2 [Source:HGNC Symbol;Acc:HGNC:9326] | 10103 | -0.331 | 0.0265 | No |
| 24 | BAAT | bile acid-CoA:amino acid N-acyltransferase [Source:HGNC Symbol;Acc:HGNC:932] | 10523 | -0.376 | 0.0322 | No |
| 25 | LYPLA2 | lysophospholipase 2 [Source:HGNC Symbol;Acc:HGNC:6738] | 10860 | -0.411 | 0.0478 | No |
| 26 | PLA2G6 | phospholipase A2 group VI [Source:HGNC Symbol;Acc:HGNC:9039] | 11128 | -0.446 | 0.0723 | No |
| 27 | FASN | fatty acid synthase [Source:HGNC Symbol;Acc:HGNC:3594] | 12194 | -0.643 | 0.0548 | No |
Table: GSEA details [plain text format]

  

Fig 2: GOMF\_THIOLESTER\_HYDROLASE\_ACTIVITY      
 Blue-Pink O' Gram in the Space of the Analyzed GeneSet

  

Fig 3: GOMF\_THIOLESTER\_HYDROLASE\_ACTIVITY: Random ES distribution      
 Gene set null distribution of ES for **GOMF\_THIOLESTER\_HYDROLASE\_ACTIVITY**
